# Transgenerational Consequences of Imidacloprid Larval Diet Contamination in the Sheep Blowfly *Lucilia sericata* (Diptera: Calliphoridae)

**DOI:** 10.3390/insects16121265

**Published:** 2025-12-12

**Authors:** Gabriela Olivares-Castro, Constanza Schapheer, Carlos Guerrero-Bosagna, Ian S. Acuña-Rodríguez, Cristian Villagra

**Affiliations:** 1 Departamento de Ciencias Ecológicas, Facultad de Ciencias, Universidad de Chile, Las Palmeras 3425, Ñuñoa, Santiago 8820808, Chile; 2 Instituto de Entomología, Universidad Metropolitana de Ciencias de la Educación, Avenida José Pedro Alessandri 774, Santiago 7760197, Chile; 3 Departamento de Ingeniería y Suelos, Facultad de Ciencias Agronómicas, Universidad de Chile, Av. Santa Rosa 11.315, La Pintana, Santiago 8820808, Chile; constanza.schapheer@uchile.cl; 4 Physiology and Environmental Toxicology Program, Department of Organismal Biology, Uppsala University, 752 36 Uppsala, Sweden; carlos.guerrero.bosagna@ebc.uu.se; 5 Centro de Ecología Integrativa (CEI), Dirección de Investigación, Vicerrectoría Académica, Universidad de Talca, Talca 3465548, Chile; ian.acuna@utalca.cl

**Keywords:** biocides, transgenerational developmental plasticity, sublethal pesticide contamination

## Abstract

Pesticides have been extensively used in agriculture, forestry, and veterinary medicine under intensive production systems. Unfortunately, pesticide pollution resulted in a significant decline in non-target organisms, for instance, in detritivores such as necrophagous insects. Even formulations proposed as less harmful alternatives, such as neonicotinoids like imidacloprid (IMI), have been demonstrated to permeate the trophic chain and trigger severe consequences on non-target species. Here, the intra- and inter-generational effects of a sublethal dose of IMI were explored in the necrophagous greenbottle fly, *Lucilia sericata* (Meigen, 1826) (Diptera: Calliphoridae). This is because it has been demonstrated that the carcasses of domestic and wild animals can be contaminated with levels of these neonicotinoids. Transgenerational effects, extending up to three generations after a focal application of the pesticide on laboratory-cultivated F_1_ flies, were investigated in this study. Morphological, demographic, and phenological features were evaluated through various analyses, including general linear mixed models (GLMM) and Haldane units analyses. Although GLMM showed no significant differences between treatments for the multiple traits observed, a significant directional microevolutionary trend of increased average imago and pupal size was identified for the IMI treatment through Haldane unit analysis. This microevolutionary change falls within the threshold of transgenerational phenotypic plasticity, a crucial mechanism for adaptive responses to environmental stressors. Among the possible explanations for this pattern, it is proposed that this is a likely consequence of the triggering of an epigenetic hormetic transgenerational change. This may contribute to explaining the development of adaptation and resistance towards pesticide formulations in a few generations after focal exposure. In addition to this idea, other possible mechanisms and consequences that explain the observed pattern are discussed. Overall, this experiment highlights the concerns of pesticide spillover consequences, even from sublethal doses of these formulations.

## Simple Summary

This study examines the effect of exposure to an initial low dose of the neonicotinoid pesticide imidacloprid on the blowfly *Lucilia sericata* to determine its potential impact on the generation exposed to the pesticide (F_1_) as well as across three subsequent generations (F_2_–F_4_) not exposed to imidacloprid. To test this, two experimental groups of flies were compared: one exposed to a single low dose of pesticide at F_1_ and a control group that was not exposed. From these two groups, flies were reared through three subsequent generations (F_2_–F_4_) without exposure to the pesticide. From F_1_ to F_4_, various traits, including size, were analyzed. When comparing the groups, differences were identified between generations and treatments that suggest microevolutionary change patterns over a small temporal scale. Based on these findings, it is proposed that further research is warranted on this topic because the presence of this pesticide in the environment may have unexpected effects on both target and non-target organisms, as well as on large-scale ecosystems.

## 1. Introduction

Pesticides are chemical substances formulated to control agricultural, forestry, and livestock-related pests by preventing, repelling, mitigating, or destroying them [1]. Due to their worldwide intensive and extensive use for over half a century, evidence of the damage pesticides cause to the environment has accumulated [2,3]. Nonetheless, there is a lack of studies considering their transgenerational effects on many organisms that are not the focus of pesticide control (non-target organisms), but are, nevertheless, affected by pesticide contamination due to the shared biological effects [4]. This is the case for detritivorous animals, such as leaf-litter invertebrates and necrophagous insects [4]. In the case of non-target necrophagous blowflies (Diptera), they feed and develop on ephemeral resources, such as decaying animal matter [5]. As carcasses can often be contaminated with pesticides, these necrophagous associates are affected by the toxic effects of these chemical compounds [4]. Adverse effects have frequently been observed in various non-target organisms with known ecosystem functions, such as carrion and coprophagous insects that are essential in the cycling of organic matter [5,6,7,8], and also pollinators that play a fundamental role in the maintenance of ecosystems and the reproduction of angiosperms [9,10,11]. All the described organisms can be exposed to pesticide contamination, with harmful consequences even in relatively low concentrations, due to agricultural [10] and veterinary applications [6,8]. The effects of pesticide pollution on non-target organisms and ecosystems are considered one of the drivers of planetary biodiversity decline [12,13,14,15].

Neonicotinoids are nitroguanidine systemic pesticides derived from nicotine that have been promoted as an alternative to the reduced specificity of other pesticides, since they act as agonists of nicotinic acetylcholine receptors (nAChRs) in insects, causing neural overstimulation [16]. In agriculture, these systemic pesticides can be applied to the seeds and diffuse throughout the rest of the plant as it grows [17], which was proposed to reduce the exposure of non-target organisms [16,18,19,20]. In veterinary applications, neonicotinoids, such as imidacloprid (IMI), are applied topically to pets and livestock to treat various acari, insect, and nematode parasites, including ticks, fleas, and roundworms, among others [21,22].

These pesticides can remain in the environment in residual amounts, which may have detrimental effects on various organisms, even in sublethal concentrations, which have been described in plants, invertebrates, and vertebrates, including humans [23,24,25,26,27]. Acetamiprid and IMI have been detected in floral and extrafloral nectar, providing sources for these systemic pesticides to permeate wild trophic chains [24,28,29,30,31]. Contamination by these compounds has been reported in various plant tissues and organs of crops and wild species, resulting in varied cytotoxicity and genotoxicity in the cultivated plants they are intended to protect [24,30,32]. As a consequence of animal exposure to neonicotinoids, it is also possible to find detectable traces of these pesticides in carcasses, which can potentially pass through other levels of the trophic chain with uncalculated consequences for ecosystems [33,34].

The detrimental effects of neonicotinoids have been observed in the development, morphology, and behavior of various insects [23,29,35,36,37]. For instance, a link has been established between neonicotinoid applications and colony collapse disorder (CCD) in honeybees [9]. Among vertebrates, exposure to neonicotinoids has revealed neurological, physiological, developmental, and reproductive problems even at sublethal doses [38,39,40,41,42,43]. Considering that these compounds have such a wide array of adverse effects, it becomes evident that there is a requirement to be more exhaustive when testing this kind of formulation and consider both experimental and field settings [29]. As a consequence, it was decided to study whether a single exposure to IMI at a sublethal dosage has a long-term effect, evaluating its potential transgenerational effects.

Among insects, neonicotinoid applications affect detritivores, such as the dipterans associated with decaying carcasses, altering their development, physiology, and contribution to decomposition [44,45,46,47]. These flies may be an unexpected casualty of pesticide application due to their association with the carrion derived from vertebrates treated or contaminated with these pesticides. However, field and controlled experiments are required to assess how much neonicotinoids, such as IMI, may affect these detritivores as non-target organisms [40]. For instance, it is unknown whether exposure to contaminated vertebrate carcasses may lead to transgenerational effects on these detritivores, to the extent that these lasting alterations could be considered microevolutionary changes, i.e., transgenerational phenotypic changes in a given species observable within a human lifespan [48,49]. In this sense, besides studying potential temporal changes through correlation and the repeated evaluation of changes in variance, a set of traits related to morphology, demography, and phenology was considered to explore the potential effects of exposure to IMI. Gingerich [50] has proposed that evolutionary change can occur on a generational timescale, as the differences observed in organisms are at the level of generations. They compound from one to another to form eventual micro and macroevolutionary trends [48]. These changes across generations can be measured in Haldane units to quantify short-term evolutionary trends. Since the aim was to evaluate transgenerational changes, these units proved to be an excellent tool for measuring observable trends [51]. Corresponding to a standardized quantification of the evolutionary change rate of continuous phenotypic traits between generations of a given organism. This metric is particularly suited for this study because it quantifies short-term evolutionary responses in units of standard deviations per generation, allowing the evaluation of phenotypic change over the narrow temporal scale relevant to this four-generation experimental design (F_1_ exposed generation, and F_2_–F_4_ subsequent non-exposed generations). Unlike traditional macroevolutionary rate metrics, Haldanes explicitly capture rapid, generational shifts, making them widely used in studies of contemporary evolution under experimental and anthropogenic pressures [52,53]. In this study, it was calculated as the difference between the mean trait values at the final and initial generations, divided by the product of the pooled phenotypic standard deviation (SD_p_) and the number of generations elapsed (Δg) [54]:

(1) $$Haldane\;\left(rate\right)=\;\frac{X_{f}-X_{i}}{SDp*\Delta g}$$

The Haldane units correspond to a change by a factor of one standard deviation per generation and were proposed by Gingerich as an alternative to *Darwin* units, which measure the changes in millions of years [51]. These units have been used to describe rates of phenotypic changes, disregarding the processes responsible for these modifications (such as selection, epigenetic change, etc.), in different species and their potential causes, including the effects of anthropogenic activity [2,48,53] or laboratory conditions [55].

Thus, under the light of current evidence, this study explores the hypothesis proposing that: If exposure to an initial sublethal dose of the neonicotinoid pesticide IMI in a necrophagous insect can affect subsequent generations of this insect, then it will be possible to distinguish microevolutionary divergences in subsequent generations after initial experimental exposure in F_1_ versus unexposed control groups.

To evaluate this idea, the effect of a sublethal dose of IMI is tested in this study on the common green bottle fly, *Lucilia sericata* (Meigen, 1826) (Diptera: Calliphoridae), a cosmopolitan necrophagous fly that can develop their larvae on decomposing carrion [56]. Therefore, due to its life cycle and ecological niche, in urban and agroecosystem settings, it is highly likely that these detritivores are frequently exposed to sub-lethal doses of pesticides [6,24].

**Figure 1:**
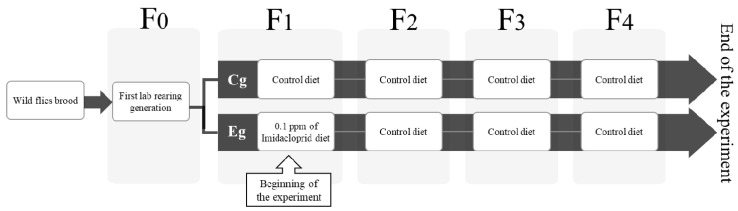
Schematic representation of *L. sericata* laboratory breeding lines. Wild flies were captured and provided with oviposition substrate. The wild F_0_ was reared from eggs laid by wild gravid females on the rearing substrate. The brood from F_0_ then became the first laboratory-reared generation, F_1_, which was separated into two treatments: control and IMI. The following three generations (F_2_ to F_4_) were fed a control diet in both groups. The experiment ended at F_4_.

The question is whether the exposure to a single sublethal dose of IMI will induce measurable phenotypic changes in *L. sericata*, and these changes persist across multiple subsequent generations that are not directly exposed to IMI. To simulate the effect of carrion contamination with this neonicotinoid pesticide, the flies were provided a single sublethal dose in their larval diet on the first laboratory-reared generation (F_1_). Then, three generations of flies (F_2_–F_4_) were reared after the original F_1_ generation exposed to IMI, and larval, pupal, and imago stages were recorded from F_1_ to F_4_ (Figure 1). GLMM, LMM, and Haldane units’ analyses were conducted on morphological, demographic, phenological, and proportional variables to evaluate the effects of the pesticide exposure and its possible transgenerational consequences through the analysis. The potential transgenerational effects of exposure to a low dose of IMI are discussed in the light of phenotypic plasticity and microevolutionary change. The relevance of the proposed transgenerational effect is highlighted considering the dynamic temporal availability of the resources where necrophagous insects develop. In that context, a “pulse” of pesticide pollution on the decaying matter may imply pervasive transgenerational consequences on necrophagous non-target organisms.

## 2. Materials and Methods

### 2.1. Insects

Laboratory colonies of *L. sericata* were established in February 2021. Flies were collected using chicken liver as bait at the municipality of Ñuñoa, Santiago de Chile, Chile (33°28′00″ S 70°36′00″ W). The adults were taxonomically determined using the key of González et al. (2017) [57]. The recollection of adults was performed every four days to reduce the risk of inbreeding. Live determined specimens of *L. sericata* were isolated and used as founders of the fly breeding lines for experimental manipulations.

### 2.2. Rearing Conditions and Diet

Four breeding lines of *L. sericata* were established under controlled temperature (26 °C) and humidity (70%), with a light cycle of 12/12 light/dark hours. After that, the adult flies were offered an oviposition substrate consisting of homogenized chicken liver (Ariztía^®^, Melipilla, Chile), which served as the base of the larval diet used in the experiment. The larval offspring obtained were designated as F_0_, and the descendants of this generation corresponded to the F_1_ exposed to IMI (Figure 1). Exploratory trials were conducted in F_1_ using three different concentrations of IMI in larval diets: 1 ppm, 0.1 ppm, and 0.01 ppm. This was prepared using a dilution of Imidacloprid (standard 37994-100MG, Sigma Aldrich, St. Louis, MO, USA) by weighing 100.0 mg of the solid standard on an analytical balance (QUIMIS^®^, Diadema, Brazil) and transferring it quantitatively to a 100 mL volumetric flask (class A), dissolving and diluting it with ultrapure water (Class III, Megaquim^®^, Valparaíso, Chile) to obtain a stock solution of 1000 ppm. From this, 5000 μL was taken with a micropipette and dissolved in a 50 mL flask to obtain a 100 ppm solution. Repeating the operation yielded a 10 ppm solution in distilled water. Then, three solutions from this initial dilution were prepared and placed in sterile Falcon tubes: 5000 µL in 50 mL of homogenized liver (1 ppm) (1); 500 µL in 50 mL (0.1 ppm) (2), and 50 µL in 50 mL (0.01 ppm) (3). Additionally, a control was established that contained only homogenized liver to which 500 µL of distilled water was added. All mixing was conducted with a vortex MX28 Biobase Bioindustry (Jinan, China) at 2500 rpm. The concentrations tested were selected based on the study of Young et al. [58], which served as a reference. 0.1 ppm was the best candidate, as it was the highest concentration with a low lethality rate.

**Figure 2:**
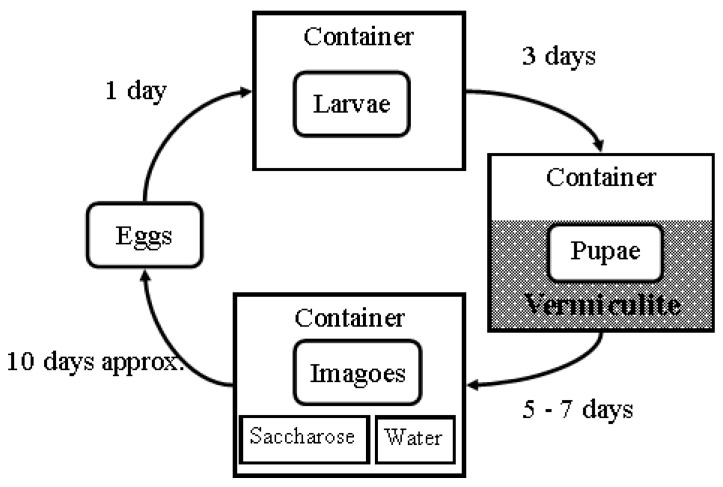
Illustration of the rearing cycle. Once oviposition on the substrate was complete, the substrate and eggs were placed in an initial plastic container, where they were allowed to hatch, feed, and grow for three days. After that, the larvae were counted and transferred to a pupation substrate composed of vermiculite in a different plastic container, where they were allowed to pupate. After five to seven days, the pupae were collected, counted, and transferred to another container with a veil cover, saccharose, and water ad libitum. The imagoes were exposed to homogenized chicken liver, allowing the females to develop their ovaries. Approximately 10 days after emergence, the flies were exposed to the homogenized substrate to facilitate oviposition.

The larvae were reared in the homogenized substrate inside plastic containers. After three days, the total number of larvae were counted, and the living ones were transferred to plastic containers filled with vermiculite as the pupation substrate. Once all larvae had pupated (which, on average, took 5 to 7 days), they were counted and the pupae were transferred to another container, where they were provided with sucrose, water, and homogenized chicken liver to facilitate the development of their ovarioles. After approximately 10 days, the chicken liver was again used as an oviposition substrate. Once females had completed oviposition, the eggs were transferred to a new container, as described in Figure 2, and the cycle continued.

**Figure 3:**
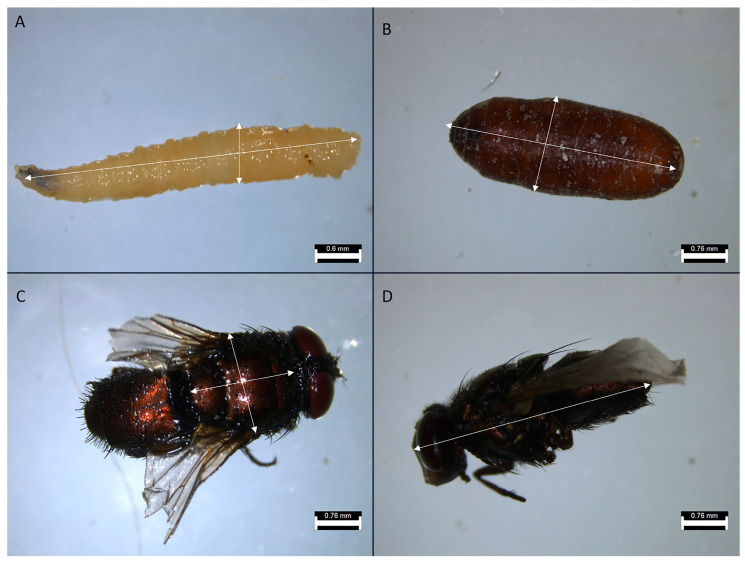
Microscopy photography of *L. sericata* live stages, the bar corresponds to the scale in millimeters. Photos are accompanied by two-headed arrows that highlight the measures taken at the different stages of development. (**A**) Larvae length and width; (**B**) Pupae length and width; (**C**) Imago thorax length and width; (**D**) Imago total length. Scale is represented in the corner of each picture.

Following the protocol described above (Figure 1 and Figure 2), six replicates of both the control and IMI treatments were performed, with each replicate originating from a different parental brood. Samples were collected from the larval, pupal, and imago stages of development to measure their size, including the total length and width of larvae, the total width and length of pupae, total body length and width, and the length of the thorax. (Figure 3). The colonies were maintained up to the fourth generation, which imposed some constraints when gathering samples. The primary constraint the survival of the colonies to complete the experiment after four generations, including the one exposed to IMI. This was necessary because, to take measurements, they had to be euthanized (See Table 1 for details). Due to these constraints, when taking larvae samples, two samples were collected from every colony, assuming a normal distribution.

Samples were collected from the smallest and largest larvae using a maximum dispersion sampling strategy [59]. The mean of the measurements from the two samples was then used for the analysis. This was performed to address the differences that may be derived from sexual dimorphism or other underlying factors [60]. It was only possible to take one sample of pupae due to the low pupation rate in some colonies. Only one adult sample was collected because it was all that was available in some colonies, and in two colonies, there were no imagoes by the F_4_ generation. The number of specimens collected was constant through the generations. The number of individuals in each stage of development was quantified considering alive third instar larvae, pupae that resulted in the emergence of imagoes, and those that failed to emerge. During each generational change some adults escaped during the process of exposure of the imagoes to the liver feeding and oviposition substrate. The time it took to progress between each development stage was also recorded.

### 2.3. Measurements and Statistics

**Figure 4:**
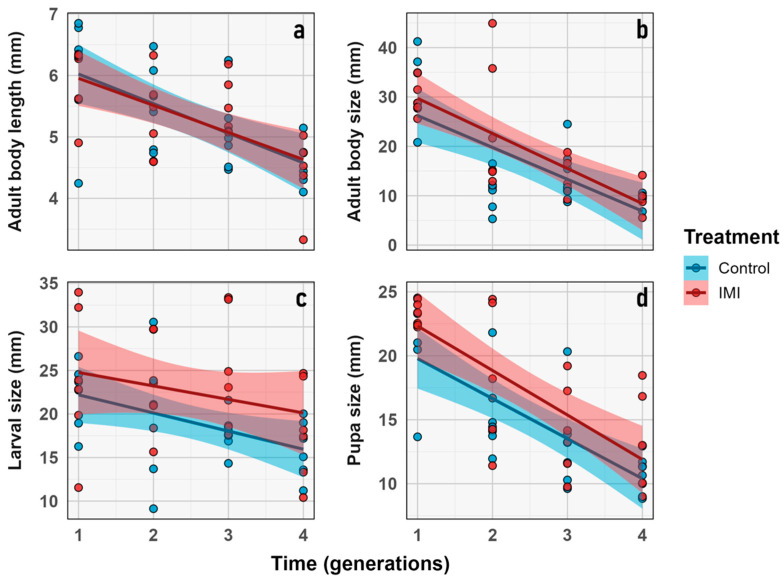
Change in morphological traits (x-axis) corresponding to: (**a**) Imago body length. (**b**) Average imago size. (**c**) Average larva size. (**d**) Pupal size, measured for *L. sericata* live stages through each generation (y axis) in control (light blue) and IMI (light red) initial exposure treatment. Dots represent the raw data dispersion, accompanied by a trend line and a standard error represented by the colored area accompanying each control and IMI treatment line.

The samples were photographed using a 14-megapixel digital camera (model H1400, Shenzhen Microtechnology, Shenzhen, China) and S-Eye software (version 1.4.1.425, YangWang Technology Co., Shenzhen, China). In Image J (version 1.53e), the resulting images were measured for insect size; a physical scale was placed next to the specimens photographed to allow for calibration and measurement. The measurements included maximum length and width in larvae and pupae, as well as maximum body length and width, and the length of the thorax in adults [61] (Figure 4). The length and width measurements in the thorax of larvae, pupae, and imagoes were then merged into single values by multiplying both values.

RStudio 2023.12.1+402 (R-Tools Technology Inc., Richmond Hill, ON, Canada, “Ocean Storm” Release (4da58325, 28 January 2024)) was used to perform Linear mixed models (LMMs) or generalized linear mixed models (GLMMs), depending on the data. Time in generations, treatment, and their interaction were considered as fixed effects, and the aleatory impact associated with the replicate as a random effect. Different variables were analyzed: morphological traits (average larval size, pupal size, average imago size, and imago body length), demographical traits (number of alive larvae and number of dead larvae), phenological traits (time to pupation and time to imago emergence), and proportional traits (percentage of emerged imagoes and lethality). Microevolutionary trends were also evaluated by calculating Haldane units, as described in the introduction of this study, which are units of measure of the evolutionary change rate of continuous phenotypic traits [51]. This metric was selected to understand how the measured variables responded to exposure to a sublethal dosage of IMI over a fixed number of generations, considering a timescale relevant to the life history of *L. sericata* [48,54]. This experimental design produced precise information on the phenotypic variation in the traits of interest in the common green bottle fly. The exact number of generations elapsed was evaluated, the temporal change associated with different treatments, key components for the study of variability patterns and the calculation of Haldane units [48]. Continuous traits (pupal and imago size) were analyzed with linear mixed models (LMM) and used to compute Haldane rates. Proportional outcomes (e.g., survival) were analyzed using generalized linear mixed models (GLMM; binomial/logit) and were excluded from Haldane calculations.

## 3. Results

### 3.1. Morphological Traits

The four morphological traits—average larval size, pupal size, average imago size, and imago body length—were analyzed using linear mixed models (LMM), with generation (time) and treatment (Control vs. IMI) as fixed effects, and experimental replicate as a random effect.

No significant effects of IMI treatment were detected on average larval size (*average_larvae_size*), nor for time, nor for their interaction. Average imago size varied significantly over time (LMM; *n* = 6; *d.f.* = 1, 9; z = 46.21; *p* < 0.0001) but was unaffected by treatment or their interaction. Imago body length (*Imago_body_lenght*) also showed a significant effect of time (LMM; *n* = 6; *d.f.* = 1, 5; z = 19.74; *p* = 0.0068), but no treatment or interaction effects (Figure 4). These results indicate that imidacloprid did not produce measurable effects on the morphological traits assessed or their temporal variation.

**Figure 5:**
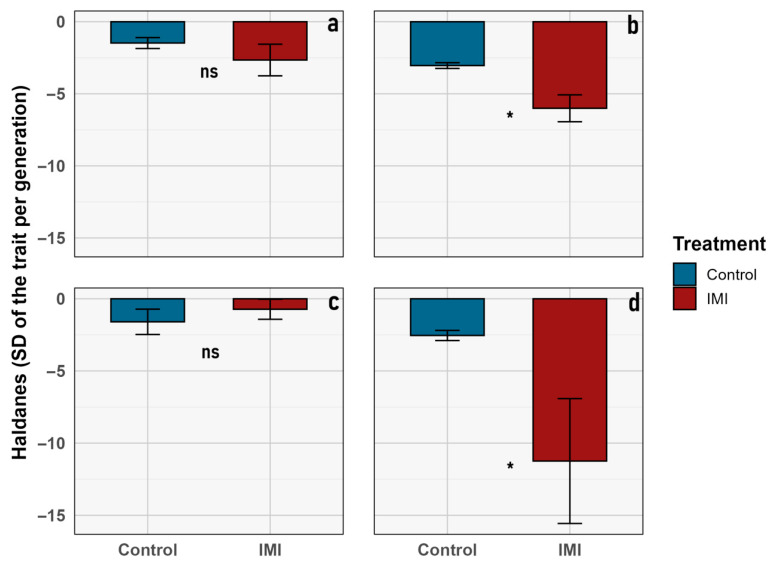
Evolutionary change rates (in Haldane units) for morphological traits of *L. sericata* across treatments. Bar plots show average values (± SD) for (**a**) imago body length, (**b**) mean imago size, (**c**) mean larval size, and (**d**) pupal size. Control and imidacloprid (IMI) treatments are shown in blue and red, respectively. Asterisks indicate statistically significant differences (*p* < 0.05), and “ns” denotes non-significant differences.

Despite the absence of significant treatment or interaction effects in the LMM analyses, the estimation of evolutionary change rates revealed a complementary pattern (Figure 5). Haldane units quantify the magnitude and direction of phenotypic change across generations, standardized by trait variance. Under this framework, all traits exhibited directional change across generations (Haldanes ≠ 0), indicating consistent temporal responses in both treatments (Figure 5). These changes appear to be in response to external factors, which will be further discussed later.

To support the calculation of generational evolutionary rates, descriptive statistics for the traits showing significant differences in Haldane values are provided (Table 2). Both *imago size* and *pupal size* exhibited marked reductions from F_1_ to F_4_ in control and insecticide-exposed lineages, consistent with directional evolutionary responses in both groups. Nevertheless, for average imago size and pupal size, the magnitude of this response differed between treatments, suggesting a possible transgenerational (microevolutionary) effect of insecticide exposure (Figure 5, Table 3).

Nevertheless, it is worth mentioning that although both analyses were designed to detect phenotypic change across generations, they capture different aspects of evolutionary response. The LMM results assess whether the observed differences among generations are statistically significant, once within-group variance is accounted for. In contrast, Haldane rates quantify the magnitude and direction of phenotypic change in standardized units per generation [54]. Consequently, Haldane values may reveal consistent directional shifts even when LMM significance is marginal, indicating evolutionary change with moderate effect sizes relative to within-lineage variability.

### 3.2. Demographic Traits

**Figure 6:**
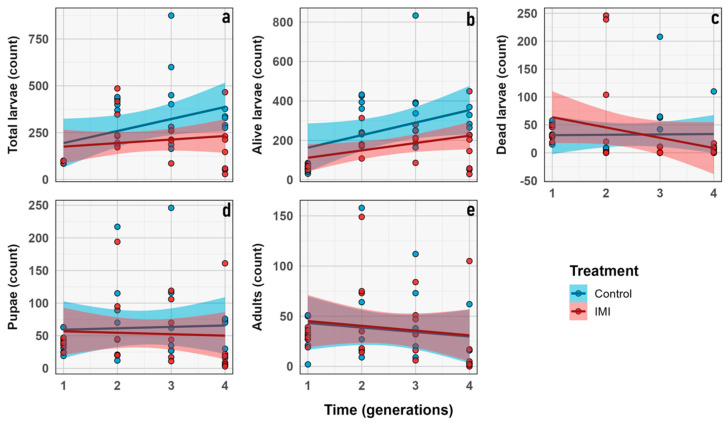
Demographic traits count (y axis) vs. generation (x axis). (**a**) Total number of larvae. (**b**) Number of living larvae. (**c**) Number of dead larvae. (**d**) Number of pupae. (**e**) Number of imagoes. Dots represent the raw data dispersion, accompanied by a trend line and a standard error represented by the colored area accompanying each line.

**Figure 7:**
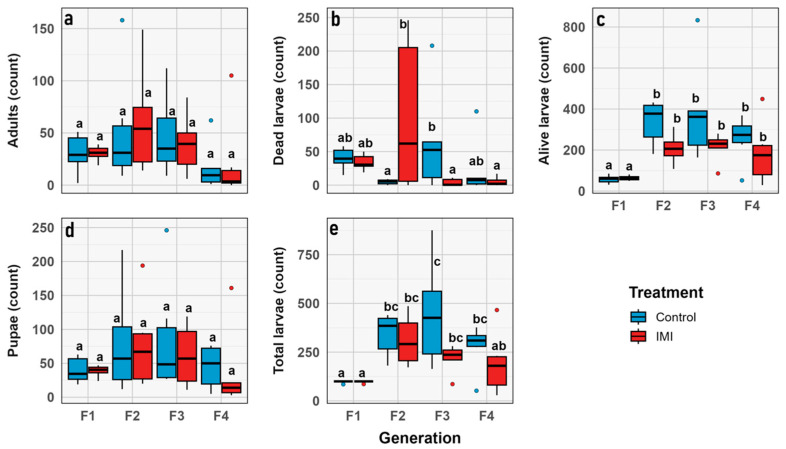
Box plot graphs showing the minimum, first quartile, median, third quartile, and maximum for demographic traits through generations (y axis) vs. combined group (x axis) correspond to: (**a**) Total number of larvae. (**b**) Number of living larvae. (**c**) Number of dead larvae. (**d**) Number of pupae. (**e**) Number of imagoes. Red corresponds to the IMI treatment, while blue represents the control treatment. Different letters within the graphs represent statistical differences between generations/treatments at *p* < 0.05. Points outside the whiskers represent outliers, defined as values beyond 1.5 times the interquartile range (IQR).

**Figure 8:**
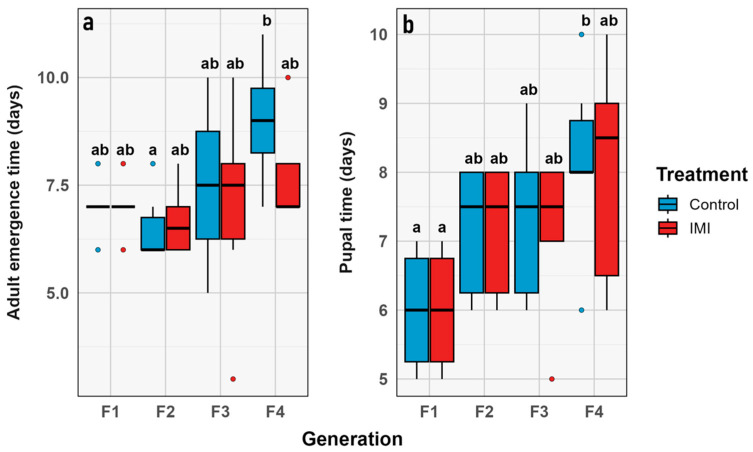
Phenological traits through generations, represented in a box plot showing the minimum, first quartile, median, third quartile, and maximum for (**a**) Time to pupation. (**b**) Time from pupation and adult emergence. Contrasting mean value vs. combined group (generation + treatment). Control in blue, IMI in rearing diet treatment in red. Red corresponds to the IMI treatment, while blue represents the control treatment. Different letters within the graphs represent statistical differences between generations/treatments at *p* < 0.05. Points outside the whiskers represent outliers, defined as values beyond 1.5 times the interquartile range (IQR).

The total number of larvae and alive larvae increased significantly across generations (GLMM; *n* = 6; *d.f.* = 1, 3; χ^2^ = 42.13; *p* < 0.0001 and *d.f.* = 1, 3; χ^2^ = 66.973; *p* < 0.0001, respectively), with no significant effects of IMI treatment or generation × treatment interaction, indicating parallel trends in both groups. Number of dead larvae showed a significant generation effect (GLMM; *n* = 6; *d.f.* = 1, 3; χ^2^ = 12.50; *p* = 0.0058) and a highly significant generation × treatment interaction (GLMM; *n* = 6; *d.f.* = 1, 3; χ^2^ = 31.75; *p* < 0.0001), suggesting that the effect of IMI on larval mortality varied across generations (Figure 6). No significant effects of generation, treatment, or their interaction were observed for pupae or adults, indicating relative stability in these stages. Overall, larval stages were more sensitive to initial IMI exposure, while pupal and adult stages were largely unaffected (Figure 7 and Figure 8).

### 3.3. Phenological Traits

**Figure 9:**
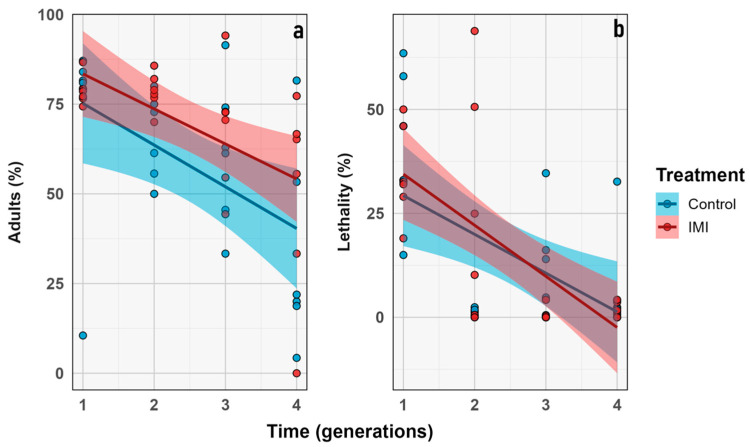
Proportional traits IMI treatment (red) vs. control (blue). Percentage (y axis) vs. generation (x axis). (**a**) Percentage of imagoes that emerged. (**b**) Percentage of lethality. Dots represent the raw data dispersion, accompanied by a trend line and a standard error represented by the colored area accompanying each line.

Analysis of the two phenological traits in *L. sericata* revealed consistent outcomes. Time to pupation (*time_p*) did not differ significantly across generations, treatments, or their interaction, suggesting that neither evolutionary time nor IMI exposure affected larval development to pupation. Likewise, time to imago emergence (*time_a*) also exhibited no significant variation relative to the experimental factors (Figure 9).

### 3.4. Proportional Traits

**Figure 10:**
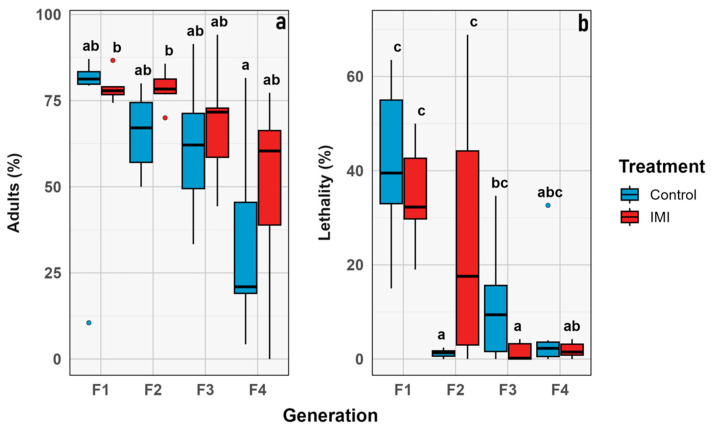
Box plot graph for proportional traits compared through generations. Intrinsic value (y axis) vs. combined group (x axis): (**a**) Percentage of imagoes that emerged. (**b**) Percentage of lethality. Blue corresponds to control, and red to IMI treatment. Different letters within the graphs represent statistical differences between generations/treatments at *p* < 0.05. Points outside the whiskers represent outliers, defined as values beyond 1.5 times the interquartile range (IQR).

The percentage of emerged imagoes and lethality were analyzed over generations. Emergence decreased significantly over time (GLMM; *n* = 6; estimate = −0.22; z = −2.38; *p* = 0.0170), but no other significant effects of IMI treatment or time × treatment interaction were detected, indicating that the decline was independent of insecticide exposure. Similarly, lethality increased over time (GLMM; *n* = 6; estimate = −0.51; z = −2.39; *p* = 0.0171), and the main effect of treatment was not significant. Categorical analysis by generation showed the highest lethality in F_1_ for both treatments (control and pesticide), which was statistically significant compared to later generations. In contrast, the percentage of emerged adults did not differ significantly within treatments across generations (*p* = 0.06). However, variability in emergence increased in later generations, possibly reflecting a more heterogeneous population response to the experimental conditions or insecticide treatment (Figure 9 and Figure 10).

## 4. Discussion

The experimental design compares the effects of an initial F_1_ generation of *L. sericata* exposed to a single sublethal dosage of IMI versus a non-exposed F_1_ control group, which were subsequently evaluated over generations (F_2_–F_4_) (Figure 1) in an allochronic approximation [54]. Although the GLMM analysis revealed no significant differences between treatments for the various traits observed, a significant microevolutionary trend was identified through Haldane unit analysis in the average imago size and pupal size. As mentioned above (Table 3, Figure 6), the Haldane units mean is significantly higher in the IMI treatment than in the control group for those two traits, which is consistent with previous studies that describe faster rates of change under anthropogenic disturbances [48,53]. These higher Haldane rates suggest a transgenerational shift toward larger body size under chemical stress, consistent with compensatory plasticity or early-stage adaptive responses to sublethal acute early-life imidacloprid exposure. Such patterns imply that anthropogenic stressors can elicit measurable transgenerational phenotypic plasticity in key morphological traits within only a few generations [48,52,53,55], reflecting both developmental flexibility and the potential onset of adaptive changes in the involved species.

The morphological traits that were measured (Figure 3) were chosen as a comprehensive approach to key aspects related to the physical characteristics of the samples [62], allowing for comparisons between treatments and across generations. Although the statistical analysis revealed no significant differences between treatments or generations, it was still useful to evaluate the presence or absence of morphological aberrations. Despite the treatment, no aberrations were observed during the experiment in any of the instars, unlike what has been previously described in other calliphorid flies subjected to chemicals in the early stages of development [63,64]. While the demographic parameters indicate general trends at the population level, no significant differences were observed between treatments or generations, suggesting that the treatment effect was insufficient to produce changes at this level. This study only considered the number of individuals, however the reproductive rate or average reproduction was not estimated which has been observed in previous studies [65]. The phenological traits considered in this study focused on the time elapsed between development stages, particularly from larvae to pupae and from pupae to imago emergence. The time between larval instars was not considered because it was not the focus of the study, even though it has been widely studied in the context of forensic entomology [66,67,68]. However, it has been previously described that exposure to other stressors, such as heavy metals, may affect these parameters [69]. The proportional traits evaluated were the percentage of emerging adults and larval lethality. They showed no significant difference between treatments; however, the highest lethality values were observed in the F_1_ (Figure 10), which may be attributed to experimental stress, as observed in both treatments with no significant differences. Other studies have previously described the relevance of parental generation exposure to certain stimuli, such as diet, in developing transgenerational responses in subsequent generations affecting traits like development time and imago body size in flies [70,71,72,73].

Non-target organisms are frequently exposed to low doses of pesticides that remain in the environment after their initial application [40], which can have negative consequences, as observed in various species of invertebrates [2,8,29,31,40,74,75]. Hormetic responses, which are beneficial effects of the exposure to low-level stressors in an organism [76] have also been described in insects exposed to sublethal doses of pesticides [77,78,79]. Necrophages are a particularly vulnerable group of organisms that are frequently exposed to various chemical stressors, including pesticides [8,40,44,64] and other substances [7] present in their diet, making them a relevant subject of study. In the traits observed in *L. sericata,* no trends were identified that suggested proper hormetic responses to sublethal exposure to IMI. Still, there was no clear negative response, despite observed changes between generations and treatments (Figure 5, Table 2).

The parental flies for the replicates were different. Still, for each of them, both the control and exposed treatments came from the same brood, to address the possible noise that using different parents would introduce when comparing the groups. There is nonetheless a risk that the founder effect could have occurred in some of the replicates; however, it is very unlikely that all six of them did so and followed the same patterns of development. Therefore, the presence of a founder effect is an unlikely explanation for the observed changes [80,81]. Inbreeding depression is another factor that may contribute to explaining the reduction in the number of adults and the decrease in size across generations and should be further explored; however, it is unlikely to account for the differences observed between the exposed and control groups in all replicates [82,83]. The changes observed in traits that exhibit microevolutionary tendencies are within the threshold of phenotypic plasticity, which is crucial in adaptive processes responding to external pressures and one of the bases on which further evolutionary change is built [50,84]. It is possible to hypothesize that the mechanisms underlying this pattern may involve the action of subjacent epigenetic molecular mechanisms (e.g., DNA methylation, histone modification, or RNA-related mechanisms), as the stimuli were presented only to the F_1_ generation, and the changes observed persist up to the F_4_ generation. Thus, future studies may focus on evaluating the expression of transgenerational epigenetic inheritance marks in necrophagous flies derived from parental generations exposed to pesticide contamination during larval development [85]. Another relevant aspect is that, as there are apparent differences among the treatments regarding their change rates (Table 2, Figure 5), it is impossible to differentiate between laboratory adaptation-originating changes [55] and the changes induced by direct exposure to IMI.

Additionally, another factor that must be considered is the potential impact of IMI on the gut microbiome of the treated breeding lines. The gut microbiome has been described as participating in various ways in insect resilience or susceptibility to abiotic stressors, such as pesticides [86]. This dimension of the problem is open for future research. Furthermore, the observed evolutionary rate is high, exceeding the threshold of ≤0.3 Haldane units discussed by Krieger et al. [87]. This suggests that IMI is a potent stressor, as even at sublethal dosages and with a single application, it can promote responses that are heritable and may result in macroevolutionary change on a broader timescale [55]. The transgenerational changes observed may be due to epigenetic mechanisms triggered by IMI exposure, which should be further explored in other studies. It has previously been reported that exposure to sublethal doses of pesticides may have effects that span multiple generations, which may be related to epigenetic responses [88,89,90]. While there is the possibility that the observed differences may be linked to differential mortality due to IMI exposure, the changes quantified by the Haldane analysis were directional between generations. They were followed through them up to the fourth one.

Considering the current results and those of previous studies [88,89,91], the most likely explanation is that the observed transgenerational response could be related to changes mediated by epigenetic mechanisms. A further path to develop in this line of investigation would be to study epigenetic marks and identify which, if any, epigenetic modifications can explain the observed changes in laboratory conditions and field assays.

Pesticides are toxic formulations with often a broad (and insufficiently tested) spectrum of action, inevitably affecting various non-target organisms. While their use has been regulated in some regions of the planet [92], they are still widely utilized, especially in the food-producing parts of the globe, Which, unfortunately, also encompasses the most biodiverse areas of the Earth [93].

Noxious effects of pesticide contamination have been observed in non-target insects [2,31,40,94,95]. For the past century, the scientific community has been sounding the alarm about the unexpected consequences of their use [75]. Some unintended consequences of pesticide use include adverse effects on predators, parasitoids, and pollinators, which can disrupt natural pest control and plant reproduction [75,88,89,91,96]. At the same time, the consequences of pesticide contamination in other ecological guilds have been less explored, such as the cases of detritivores and necrophagous invertebrates [5,6,7,8].

As some pesticides have been banned [97], the industry continues to develop new formulas whose ecological costs need to be evaluated [92]. Based on the evidence presented in this study, it is adequate to emphasize further the importance of assessing sub-lethal concentration effects to understand their short- and long-term consequences on non-target organisms.

## 5. Conclusions

The unwanted effects of pesticide use are inevitable and should be considered when applying them. As modern agriculture is highly dependent on these substances, it is necessary to identify the kind and amount of damage they may cause to opt for the less detrimental options. Typically, studies focus on the lethal effects of pesticides; however, it has become evident that these compounds may also have sub-lethal effects [19]. In this study, it was observed that sublethal doses of IMI on *L. sericata* can elicit changes at a microevolutionary level, reflecting the complex nature of interactions between organisms and stressors. Further research on the effects of sub-lethal doses on non-target species is needed to understand the broader consequences of pesticide use.

## Figures and Tables

**Table 1:** Number of specimens per treatment and generation.

Generation	Treatment	Replicate	Larvae	Pupae	Imagoes
F_1_	Control	1	85	19	2
		2	100	38	31
		3	100	31	27
		4	100	25	21
		5	100	63	50
		6	100	63	51
	Exposed	1	86	24	19
		2	100	45	39
		3	100	42	33
		4	100	47	36
		5	100	35	27
		6	100	39	29
F_2_	Control	1	181	12	9
		2	400	217	158
		3	370	70	35
		4	431	20	16
		5	440	115	64
		6	232	44	27
	Exposed	1	172	21	18
		2	486	89	73
		3	417	194	149
		4	347	45	35
		5	196	95	75
		6	236	20	14
F_3_	Control	1	187	35	32
		2	600	246	112
		3	450	116	73
		4	402	27	20
		5	164	27	9
		6	875	62	38
	Exposed	1	260	70	51
		2	213	119	84
		3	209	44	32
		4	281	17	16
		5	86	11	6
		6	261	106	47
F_4_	Control	1	337	5	1
		2	290	30	16
		3	377	73	16
		4	329	16	3
		5	52	70	3
		6	276	76	62
	Exposed	1	147	22	17
		2	231	9	5
		3	466	161	105
		4	213	6	2
		5	29	19	0
		6	59	3	2

**Table 2:** Mean ± SD and sample size (*n*) for traits used in Haldane rate calculations across all generations (F_1_ vs. F_4_) in control and imidacloprid-exposed lineages.

Trait	Treatment	Generation	Mean	SD	*n*
***Pupal size***	Control	F_1_	20.91	3.84	6
		F_4_	11.14	1.35	6
	Imidacloprid	F_1_	23.17	0.91	6
		F_4_	12.89	3.95	6
***Imago size***	Control	F_1_	31.78	7.38	6
		F_4_	9.34	1.47	6
	Imidacloprid	F_1_	29.41	3.31	6
		F_4_	9.55	3.08	6

**Table 3:** Evolutionary response of morphological data, Significant differences between control and IMI-treatment mean values are highlighted in bold p values. *t* = *t* statistic, *d.f.* = degrees of freedom, *p* = *p* value.

Trait	Haldane Mean Control	Haldane Mean IMI	*t*	*d.f.*	*p*
Average larvae size	−1.60	−0.72	−1.90	9.50	0.0876
Pupae size	−2.54	−11.24	4.90	5.06	0.0043
Average imago size	−3.04	−6.01	6.94	4.36	0.0017
Imago body length	−1.48	−2.66	2.29	4.80	0.0729

## Data Availability

The raw data supporting the conclusions of this article will be made available by the authors on request.
